# Platinum-based adjuvant therapy was efficient for triple-negative breast cancer: a meta-analysis from randomized controlled trials

**DOI:** 10.1080/21655979.2022.2115616

**Published:** 2022-10-24

**Authors:** Kaigang Xie, Xuanlei Ren, Xiaoming Hong, Shuiyin Zhu, Dongjie Wang, Xiaoming Ye, Xiaoting Ren

**Affiliations:** Department of General Surgery, the Yinzhou Second Hospital, Ningbo, China

**Keywords:** triple-negative breast cancer, platinum, neoadjuvant therapy, survival

## Abstract

Triple-negative breast cancer (TNBC) is the most aggressive breast cancer. Neoadjuvant chemotherapy was widely accepted for treating TNBC. This systematic review and meta-analysis aimed to evaluate the efficacy, safety, and survival benefit of platinum-based adjuvant therapy (PBAT) in treating TNBC. The keywords were searched in Medline, Embase, Pubmed, and Cochrane Library database up to July 24, 2022. All the randomized control trials (RCTs) comparing PBAT and non-PBAT in treating TNBC were included in our study. The pathological complete remission (pCR) and complications were compared by odds ratio (OR) and 95% confidence intervals (CIs). The overall survival (OS) and relapse-free survival (RFS) were compared by hazard ratio (HR) and 95% CIs. A total of 19 RCTs were included in our meta-analysis, among which 2,501 patients were treated with PBAT and 2,290 with non-PBAT. The patients treated with PBAT combined a significantly higher pCR rate compared to those patients treated with non-PBAT (49.8% versus 36.4%, OR = 1.27, 95%CI = 1.14–1.43, P < 0.001). Besides, patients treated with PBAT had a significantly better RFS (HR = 0.78, 95%CI = 0.63–0.95, P = 0.016), but not in OS (HR = 0.84, P = 0.304). Although the occurrence of neutropenia and nausea were slightly different between the PBAT group (51.5% and 24.4%) and the non-PBAT group (47.0% and 29.4%), the complications were acceptable in the two treatments groups. Our results demonstrated that TNBC patients treated with PBAT could achieve a higher pCR rate and better RFS benefit without a higher complication rate.

Highlights

- Platinum-based adjuvant therapy provided a higher pCR rate for TNBC.
- Platinum-based adjuvant therapy prolonged the RFS but without prolongingthe OS.
- Neutropenia and nausea rate was different between group PBAT and non-PBAT.

## Graphical abstract

**Figure:**
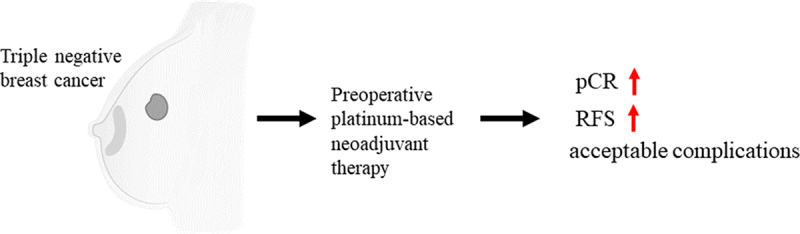

## 1. Introduction

Breast cancer (BC) has become the first malignant tumor that threatens the health of women in the world [1]. Among all malignant tumors, BC is considered to be one of the main causes of death in postmenopausal women, accounting for about 23% of all malignant tumor deaths [2]. Triple-negative breast cancer (TNBC) is a special type of breast cancer with all negative expressions in estrogen receptor (ER), progesterone receptor (PR), and human epidermal growth factor receptor-2, HER-2) [3]. TNBC is one of the most aggressive types of BC, with biological characteristics such as rapid recurrence, short disease-free survival, and a high risk of distant metastasis [3,4].

Due to its special immunohistochemical characteristics, endocrine and anti-HER-2 targeted therapies are ineffective. Currently, chemotherapy is still the most widely used treatment for TNBC. Among them, neoadjuvant chemotherapy (NAC) has the characteristics of reducing clinical staging, eliminating micro-metastases, minimizing postoperative complications, and improving the success rate of surgery. Thereafter, NAC improves survival outcomes by achieving pathological complete remission (pCR) after initial treatment [5]. Generally, NAC included single anthracycline or taxanes, anthracyclines combined with taxanes, and other regimens (methotrexate, fluorouracil, and capecitabine). Studies have shown that compared with other subtypes of BC, women with TNBC are more sensitive to the initial anthracyclines and taxanes, plan with a clinical response rate of up to 85% and a pathological pCR of 30–40% [6].

Platinum drugs (such as carboplatin and cisplatin) inhibit the replication of the DNA double-strands by cross-linking with the tumor cells’ DNA double-strands, which leads to the death of tumor cells [7]. BRCA gene mutations in tumor cells have DNA damage repair barriers. This special mechanism of action makes platinum particularly sensitive in cancer cells with DNA repair defects [7,8]. Although many clinical studies have shown that the addition of platinum in NAC can significantly increase the pCR rate of TNBC, these results are mostly obtained from retrospective, small sample-size non-randomized controlled trials (RCTs) [9–12].

More recently, several RCTs were designed for comparing the efficacy of the platinum-based neoadjuvant therapy (PBAT), which might enhance the efficacy of PBAT in treating TNBC. Thus, in this study, we conducted a systematic review and meta-analysis to discuss the efficacy of PBAT in treating TNBC from those high-quality RCTs. We evaluated the benefits in treatment response and survival benefits and assessed the safety of PBAT in treating TNBC. This study provided a shred of strong evidence for clinical guidance in TNBC treatment.

## 2. Methods

### 2.1 Search strategy

This study was performed following the preferred reporting items for systematic review and meta-analysis (PRISMA) guidelines [13]. The local ethical approval could be waived due to the design of this study.

This systematic review and meta-analysis were designed for evaluating the efficacy of PBAT in treating TNBC. All literature was searched in Ovid Medline, Embase, PubMed, and Cochrane Library database up to September 20^th^, 2020. The databases above were re-searched until July 24, 2022 to include the latest updated studies. Google Scholar and related oncological websites were also searched for finding gray literature. The keywords and medical sub-headings (MeSH) terms were designed and listed in brief as follows: ‘neoadjuvant’, ‘platinum’ included ‘carboplatin’, ‘cisplatin’, ‘nedaplatin’, ‘oxaliplatin’, ‘lobaplatin’, and ‘triple-negative breast neoplasms’ OR ‘cancer’. All the results were downloaded and imported into Endnote for further duplication deletion and literation screening. The study was approved by the ethics committee of Yinzhou Second Hospital (Approval number: #2021016).

### 2.2 Inclusion and exclusion criteria

The studies fulfilled the following inclusion criteria were included in our study: 1) the studies compared the PBAT and non-PBAT in treating TNBC, in which at least two comparative groups were contained in one study; 2) the study was an RCT; 3) the pathological examination and immunohistochemistry confirmed the diagnosis of TNBC; 4) pCR was assessed for the treatment outcome. The exclusion criteria were as follows: 1) the study reported as a case report, retrospective cohort, or case-control; 2) data could not be extracted from the original articles; 3) the cancer was not diagnosed as TNBC; 4) no comparison between PBAT and non-PBAT; 5) the study was reported in languages other than English. The reviews, other related meta-analyses, comments, and editorial or conference abstracts were read for the further inclusion of the original studies.

### 2.3 Literature screening and data extraction

Two investigators independently screened the titles and abstracts based on the inclusion and exclusion criteria. The full texts were further evaluated if the abstracts and titles could not determine for the final inclusion of the studies. The third investigator was adapted for discussion if there were any disagreements existed in the literature screening. After that, two investigators independently extracted the data from the original study. The data was imported into a standard Excel form. The extracted data were listed as follows: 1) author, publish year, institution, recruitment period, study type, diagnosis; 2) the characteristics of the treatment approach and group, such as patient sample, age, gender, menopausal status, histological grade; 3) pCR, complication outcome, and survival outcome.

### 2.4 Quality assessment

Two investigators independently assessed the quality of the included studies. For the RCTs, the Newcastle-Ottawa Quality Assessment Scale (NOS) was used with a high quality of 6–9, whereas low quality was scored as 0–5 [14].

### 2.5 Statistical analysis

The category variables such as pCR and complications were compared using odds ratio (OR) and 95% confidence intervals (CIs). The hazard ratio (HR) and 95% CIs associated with overall survival (OS) and relapse-free survival (RFS) were extracted from either univariate or multivariate Cox regression analyses. If the HR was not reported in the analysis, we calculated the time-to-event data through the survival curve based on Tierney’s method [15]. The likelihood χ^2^ test and I^2^ statistics were used for detecting heterogeneity across studies (I [2] ≥50% indicating the presence of heterogeneity). When heterogeneity existed among studies, the random effect model was conducted for calculating the pooled HRs, otherwise, the fixed effect model was adopted. P value less than 0.05 was regarded as statistically significant. All statistical analyses were performed by Stata 15.0 software (Stata Corporation, TX, USA).

## 3. Results

### 3.1 Literature screening

**Figure 1.**
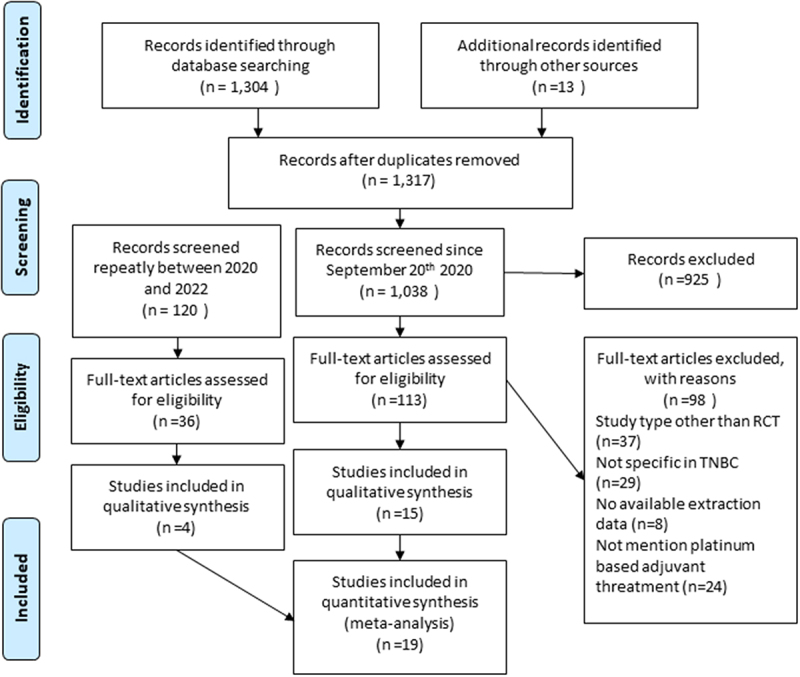
The flowchart of literature screening.

A total of 1,317 studies were identified through a systematic search from related databases and gray literature. After deleting the duplicated studies, 1,038 studies were screened from the titles and abstracts. Besides, 120 studies were re-searched in the second period of this study, and the full-text abstracts were assessed from 149 studies according to the inclusion and exclusion criteria. Finally, 19 studies were included in our systematic review and meta-analysis ^10−12,^ [16–31]. The flowchart of the literature screening is shown in Figure 1.

### 3.2 The characteristics of included studies

The characteristics of included studies are shown in Table 1 and Table 2. A total of 19 RCTs compared the PBAT and non-PABT NACs in treating TNBC patients. Seventeen studies used the standard chemotherapy as the control group, which used anthracyclines and taxanes. The rest two studies added the bevacizumab in the control group. Ten studies were designed as Phase II RCTs. Three studies were designed as Phase III studies. The studies were reported from China, Germany, Israel, Switzerland, the USA, Japan, and Spain, which recruit patients from 2003 to 2021.

**Table 1.** The characteristics of included studies.

Author	Year	Recruitment year	Country	study type	BRCA mutation	treatment	NOS
Zhang, L.	2022	2016–2019	China	RCT	NS	epirubicin, docetaxel, and cyclophosphamide vs. docetaxel and carboplatin	7
Yan, W.	2022	2014–2019	China	RCT	NS	docetaxel, epirubicin ± lobaplatin	7
Mayer, I. A.	2021	2016–2021	USA	RCT Phase III	NS	capecitabine vs. Platinum	8
Pohl-Rescigno, E.	2020	2014–2016	Germany	RCT	NS	epirubicin, paclitaxel, and cyclophosphamide vs. paclitaxel, doxorubicin and carboplatin	5
Zhang, J.	2020	2003–2015	China	RCT	Yes	Anthracycline taxane ± carboplatin	7
Du, F.	2020	2009–2015	China	RCT Phase II	Yes	Epirubicin, cyclophosphamide followed by taxanes (paclitaxel or docetaxel) vs. carboplatin, taxanes (paclitaxel or docetaxel)	6
Schneeweiss, A.	2019	2014–2016	Germany	RCT Phase III	NS	Epirubicin, paclitaxel, and cyclophosphamide vs. paclitaxel, doxorubicin and carboplatin	8
Wu, X.	2018	2014–2017	China	RCT Phase II	NS	Docetaxel, epirubicin ± lobaplatin	8
Su, Y. W.	2018	2004–2010	China	RCT	Yes	Anthracycline taxane ± carboplatin	6
Sella, T.	2018	2013–2017	Israel	RCT Phase II	Yes	Doxorubicin and cyclophosphamide ± paclitaxel and carboplatin	7
Loibl, S.	2018	NA	Germany	RCT Phase III	Yes	Paclitaxel, doxorubicin, cyclophosphamide ± carboplatin\veliparib	8
Gluz, O.	2018	2013–2015	Germany	RCT Phase II	NS	Paclitaxel and carboplatinum vs. paclitaxel and gemcitabine	8
Vetter, M.	2017	2004–2014	Switzerland	RCT	Yes	Anthracycline taxane ± carboplatin	8
Zhang, P.	2016	2006–2012	China	RCT Phase II	Yes	Anthracycline taxane ± carboplatin	8
Rugo, H. S.	2016	2010–2012	USA	RCT Phase II	Yes	Paclitaxel, doxorubicin, cyclophosphamide ± carboplatin\veliparib	7
Sikov, W. M.	2015	2009–2012	USA	RCT Phase II	Yes	Anthracycline ± bevacizumab\platine	8
von Minckwitz, G.	2014	2011–2012	Germany	RCT Phase II	NS	Anthracycline, bevacizumab ± platine	8
Ando, M.	2014	2010–2011	Japan	RCT Phase II	NS	Anthracycline taxane ± carboplatin	8
Alba, E.	2012	2007–2010	Spain	RCT Phase II	Yes	Anthracycline taxane ± carboplatin	7

Abbreviation: RCT: randomized control trial; NOS: the Newcastle-Ottawa Quality Assessment Scale; NS: not specific

**Table 2.** The characteristics of patients in the included studies.

Author	Year	Group	sample	age	Premenopausal	Postmenopausal	Histological grade I	Histological grade II	Histological grade III
Zhang, L.	2022	Non platinum-based neoadjuvant treatment	44	NG	NG	NG	NG	NG	27 (61)
		Platinum-based neoadjuvant treatment	44	NG	NG	NG	NG	NG	29 (66)
Yan, W.	2022	Non platinum-based neoadjuvant treatment	101	NG	NG	NG	18 (18)	64 (63)	19 (19)
		Platinum-based neoadjuvant treatment	99	NG	NG	NG	16 (16)	62 (63)	21 (21)
Mayer, I. A.	2021	Non platinum-based neoadjuvant treatment	160	52 (26–76)	NG	NG	15 (9)	101 (63)	35 (22)
		Platinum-based neoadjuvant treatment	148	52 (27–72)	NG	NG	21 (14)	83 (56)	77 (52)
Pohl-Rescigno, E.	2020	Non platinum-based neoadjuvant treatment	34	NG	NG	NG	NG	NG	NG
		Platinum-based neoadjuvant treatment	35	NG	NG	NG	NG	NG	NG
Zhang, J.	2020	Non platinum-based neoadjuvant treatment	155	NG	NG	NG	NG	NG	NG
		Platinum-based neoadjuvant treatment	133	NG	NG	NG	NG	NG	NG
Du, F.	2020	Non platinum-based neoadjuvant treatment	154	48.0 (9.8)	90 (58)	64 (42)	54 (35)	83 (54)	17 (11)
		Platinum-based neoadjuvant treatment	154	48.8 (9.9)	94 (61)	60 (39)	62 (40)	83 (54)	9 (6)
Schneeweiss, A.	2019	Non platinum-based neoadjuvant treatment	470	48.0 (23.0e76.0)	288 (61)	182 (39)	155 (33)	202 (43)	35 (7)
		Platinum-based neoadjuvant treatment	475	48.0 (21.0e76.0)	290 (61)	185 (39)	172 (36)	196 (41)	31 (7)
Wu, X.	2018	Non platinum-based neoadjuvant treatment	63	NG	NG	NG	6 (10)	43 (68)	14 (22)
		Platinum-based neoadjuvant treatment	62	NG	NG	NG	5 (8)	36 (58)	21 (34)
Su, Y. W.	2018	Platinum-based neoadjuvant treatment	25	51.1 (31.1–70.2)	NG	NG	5 (20)	NG	NG
		Non platinum-based neoadjuvant treatment	104	53.4 (21.6–79.9)	NG	NG	23 (22)	NG	NG
Sella, T.	2018	Platinum-based neoadjuvant treatment	76	43.3 (10.4)	NG	NG	0 (0)	47 (62)	29 (38)
		Non platinum-based neoadjuvant treatment	43	42.3 (9.5)	NG	NG	2 (5)	22 (51)	19 (44)
Loibl, S.	2018	Platinum-based neoadjuvant treatment	316	51 (41–59)	NG	NG	NG	NG	NG
		Platinum-based neoadjuvant treatment	160	49 (40–57)	NG	NG	NG	NG	NG
		Non platinum-based neoadjuvant treatment	158	50 (42–59)	NG	NG	NG	NG	NG
Gluz, O.	2018	Non platinum-based neoadjuvant treatment	178	NG	NG	NG	NG	NG	NG
		Platinum-based neoadjuvant treatment	146	NG	NG	NG	NG	NG	NG
Vetter, M.	2017	Non platinum-based neoadjuvant treatment	54	51 (26–82)	NG	NG	22 (41)	30 (56)	2 (4)
		Platinum-based neoadjuvant treatment	29	53 (38–71)	NG	NG	12 (41)	14 (48)	3 (10)
Zhang, P.	2016	Non platinum-based neoadjuvant treatment	47	48 (24–73)	29 (62)	18 (38)	NG	16 (34)	31 (66)
		Platinum-based neoadjuvant treatment	44	46 (24–65)	30 (68)	14 (32)	NG	15 (34)	29 (66)
Rugo, H. S.	2016	Platinum-based neoadjuvant treatment	39	47.5 (24–71)	NG	NG	NG	NG	NG
		Non platinum-based neoadjuvant treatment	21	48.5 (27–70)	NG	NG	NG	NG	NG
Sikov, W. M.	2015	Non platinum-based neoadjuvant treatment	107	NG	NG	NG	NG	NG	NG
		Non platinum-based neoadjuvant treatment	105	NG	NG	NG	NG	NG	NG
		Platinum-based neoadjuvant treatment	111	NG	NG	NG	NG	NG	NG
		Platinum-based neoadjuvant treatment	110	NG	NG	NG	NG	NG	NG
von Minckwitz, G.	2014	Non platinum-based neoadjuvant treatment	157	47 (21–78)	NG	NG	NG	NG	NG
		Platinum-based neoadjuvant treatment	158	48 (21–75)	NG	NG	NG	NG	NG
Ando, M.	2014	Non platinum-based neoadjuvant treatment	88	47 (30–69)	60 (68)	28 (32)	16 (18)	29 (33)	43 (49)
		Platinum-based neoadjuvant treatment	91	47 (30–70)	54 (59)	37 (41)	13 (14)	35 (38)	43 (47)
Alba, E.	2012	Platinum-based neoadjuvant treatment	46	47 (27–70)	33 (72)	13 (28)	1 (2)	9 (20)	36 (78)
		Non platinum-based neoadjuvant treatment	47	47 (28–75)	29 (62)	19 (40)	2 (4)	13 (28)	33 (70)

Abbreviation: NG: not given

A total of 4,791 patients were involved in our meta-analysis, among which, 2,501 patients were treated with PBAT and 2,290 patients were treated with non-PBAT. The median age ranged from 43.3 to 53 years old in the PBAT group, while 42 to 53.4 years old in the non-PBAT group. 64% of the female patients were in premenopausal status in the PBAT group and 61% in the non-PBAT group. In terms of histological grade, 19%, 43%, and 39% of the patients were diagnosed with grades I, II, and III in the PBAT group, respectively. 19%, 49%, and 34% were diagnosed as I, II, and III in the non-PBAT group, respectively.

### 3.3 Quality assessment

The quality assessment among studies is described in Table 1. Ten studies were scored as eight and six studies were scored as 7 which could be regarded as high quality.

### 3.4 The comparison of pCR in two treatment groups

**Figure 2.**
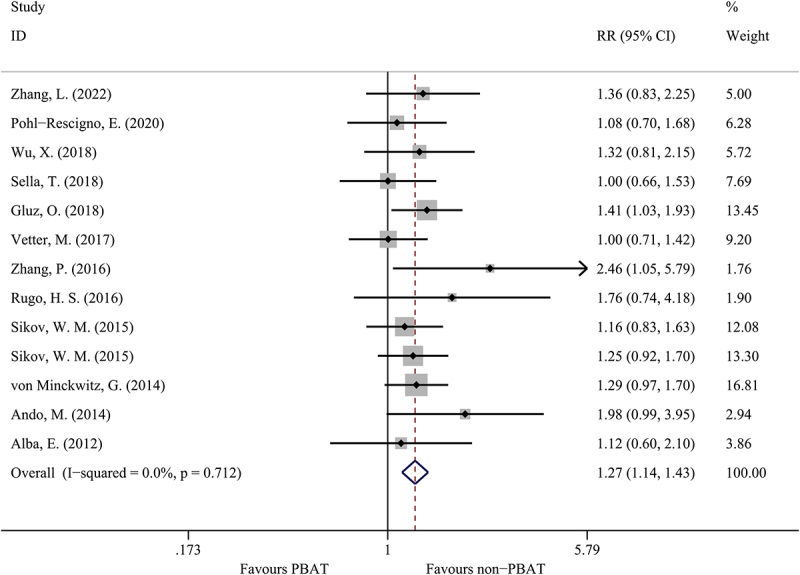
The comparison of pCR in PBAT and non-PBAT in treating TNBC.

Fourteen studies evaluated the pCR rates in TNBC patients, as shown in Figure 2. The patients treated with PBAT combined a significantly higher pCR rate compared to those patients treated with non-PBAT (49.8% versus 36.4%, OR = 1.27, 95%CI = 1.14–1.43, I [2]=0.0%, P < 0.001). Among those studies which only used standard chemotherapy (anthracyclines and taxanes) as adjuvant treatment. The patients treated with PBAT still had a higher pCR rate compared to those patients treated with non-PBAT (OR = 1.30, 95%CI = 1.12–1.51, I [2]=0.0%, P < 0.001).

### 3.5 The comparison of drug-related complications in two treatment groups

**Figure 3.**
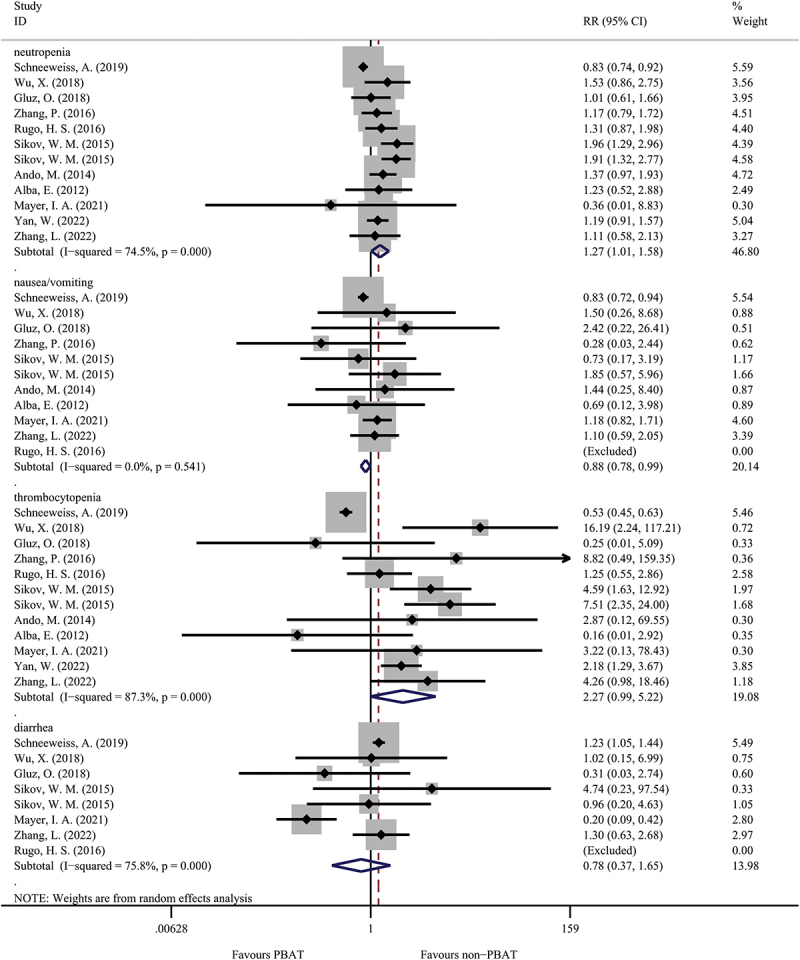
The comparison of drug-related complications in PBAT and non-PBAT in treating TNBC.

A total of eight studies evaluated the drug-related complications between PBAT and non-PBAT groups (Figure 3). The patients treated with PBAT had a higher percentage of neutropenia than patients treated with non-PBAT (51.5% versus 47.0%, OR = 1.27, 95%CI = 1.01–1.58, I [2]=74.5%, random effect model, P = 0.037). On the contrary, in terms of nausea and vomiting, the occurrence was slightly higher in non-PBAT group than in the PBAT group (29.4% versus 24.4%, OR = 0.88, 95%CI = 0.78–0.99, I [2]=0.0%, P = 0.037). However, there was no significant difference in comparing thrombocytopenia in the two treatment groups (OR = 2.27, 95%CI = 0.99–5.22, I [2]=87.3%, random effect model, P = 0.054). Similarly, there was no significant difference in the occurrence of diarrhea between the PBAT group than non-PBAT group (22.4% versus 25.6%, OR = 0.78, 95%CI = 0.37–1.65, I [2]=75.8%, P = 0.524).

### 3.6 The comparison of survival rates in two groups

**Figure 4.**
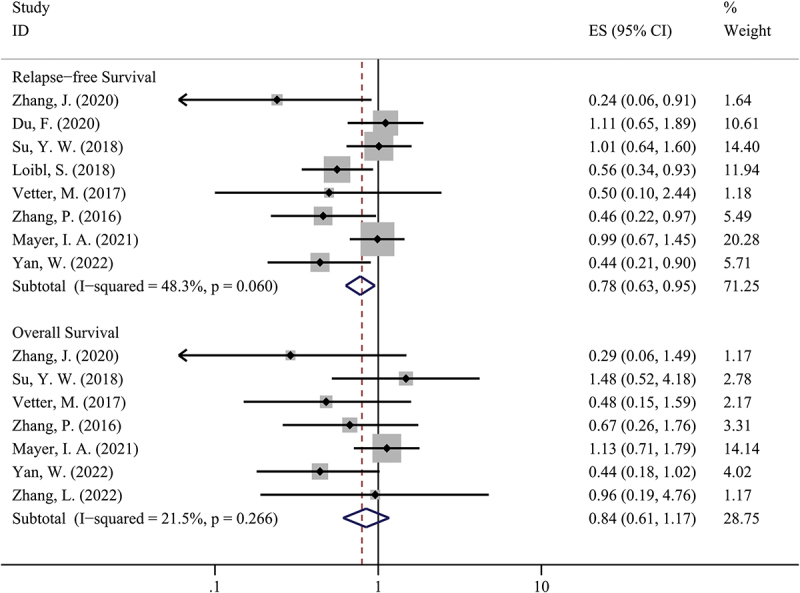
The comparison of relapse-free survival and overall survival in PBAT and non-PBAT in treating TNBC.

We used the ‘time-to-event’ approach to compare the survival outcome in two groups, which was shown in Figure 4. Our study suggested that patients treated with PBAT could have a better RFS compared to patients treated with non-PBAT (HR = 0.78, 95%CI = 0.63–0.95, I [2]=48.3%, P = 0.016). However, there was no significant difference between the two groups in terms of OS (HR = 0.84, 95%CI = 0.61–1.17, I [2]=21.5%, P = 0.304).

There were nine studies focused on the BRCA mutation in TNBC when comparing PBAT and non-PBAT. Similarly, we found that the BRCA mutated patients treated with PBAT combined a significantly higher pCR rate compared to those patients treated with non-PBAT (OR = 1.20, 95%CI = 1.02–1.41, I [2]=5.3%, P = 0.030). Besides, due to all the studies comparing RFS and OS focused on the BRCA mutated TNBC patients, the RFS and OS were similar to the results reported above.

## 4. Discussion

In our study, we compared the pCR, complication, and survival rate in patients treated with PBAT, which demonstrated that PBAT could provide a higher pCR with acceptable drug-related complications. This is the first study using the ‘time-to-event’ method to compare the survival outcome, which suggested that PBAT could prolong RFS, however, no statistical improvement in OS. As far as we concerned, this is the largest sample-size meta-analysis including only RCTs ever since. We analyzed both the pCR and survival benefit, as well as the safety of the PBAT.

TNBC is a type of highly invasive BC, which is usually accompanied by highly invasive clinical biological behaviors and most of the unique metastatic patterns [32]. Dent et al. conducted a long-term follow-up of a large number of TNBC cases in a hospital and showed that the mid-term survival rate (4.2 years) of patients with TNBC is higher than that of non-TNBC cases. The OS (6 years) is short, and all TNBC patients die within 10 years [3]. Compared to non-TNBC, studies have found that TNBC is more prone to distant metastasis and recurrence (20.4%–20.4%). These studies indicate that the biological behaviors of TNBC are different from other types of BC [33]. Many studies have confirmed that TNBC is likely to be concealed by mammography and color Doppler ultrasound imaging, and clinically [3]. The relationship between tumor size and lymph node metastasis is not strongly correlated in TNBC. Its invasiveness is significantly higher than other subtypes of BC [34]. TNBC is also prone to occur in ductal tissues, is highly malignant, and has a high proliferation and mitotic rate. Lara-Medina et al. demonstrated that TNBC had a higher risk of local recurrence, and low disease-free survival and survival rate [35].

The currently most effective adjuvant regimens for TNBC are still not fully defined in both early and advanced diseases, but chemotherapy regimens using dose-dense or metronomic polychemotherapy are among the most effective. Targets of TNBC treatment strategies include DNA repair complexes (platinum compounds and taxanes), P53 (taxanes), cell proliferation (anthracycline-containing regimens), and targeted therapy. In addition, several studies have attempted to determine the additional benefit of adding novel chemotherapeutic agents in combination with standard chemotherapy such as anthracyclines, paclitaxel, antimetabolites, platinum agents, and novel microtubule stabilizers [36–38]. TNBC is biologically aggressive, with some reports showing that patients respond better to chemotherapy than other types of BC. NAC provides a model for rapid assessment of treatment effects, with the benefit of improved patient benefit and a shorter duration of time compared to conventional adjuvant chemotherapy [31]. In addition, NAC provides an opportunity to determine tumor response to chemotherapy in vivo. The association of pCR with survival outcomes has also been observed in NAC studies; therefore, pCR is considered an important endpoint in clinical trials evaluating the efficacy of NAC [10].

NAC regimens based on anthracyclines and taxanes are currently an important strategy for the treatment of TNBC. Taxanes are very important drugs for the treatment of TNBC, but they do not show special efficacy in the treatment of non-TNBC. The efficacy of the treatment remains controversial. The chemosensitivity of tumors harboring p53 mutations is a controversial feature of TNBC, as resistance to anthracycline chemotherapy has been reported in p53-mutated BCs. Capecitabine is generally used in the metastatic treatment of advanced BC [25]. It is highly selective for cancer cells and is converted into 5-fluorouracil with antitumor activity by thymidine phosphorylase in tumor tissue, to play an anti-tumor effect. The hand-foot syndrome occurs in almost half of patients taking capecitabine. Studies have found that taxanes can increase TPase activity, thus increasing the therapeutic effect of capecitabine [17].

The mutation rate of the TP53 gene in BC is around 30%, and the mutation rate in TNBC can reach 54% [39]. As a tumor suppressor gene, wild-type p53 promotes cell malignant transformation and hinders cell apoptosis after mutation. Wild-type p53 is closely related to the signal transduction that PBATs induce tumor cell apoptosis. Highly expressed mutant p53 will inhibit wild-type p53, leading to drug resistance in tumor cells [40]. Many clinical studies have shown that patients with p53 mutations in TNBC are not sensitive to PBAT drugs, and the 5-year survival rate is reduced, which is an indicator of poor prognosis for TNBC. TP63 is a member of the p53 family. In TNBC, TP63 and TP73 are expressed cooperatively, and TP63 can antagonize the transcriptional activity of TP73 and inhibit its expression [41]. Leong et al. reported that cells with p63 gene mutations have a higher sensitivity to cisplatin [42]. Therefore, cisplatin can regulate the activity of p73 directly and indirectly. The direct effect is by inducing the transcription activity of the p73 gene, and the indirect effect is by reducing its antagonist p63 [42]. Rocca conducted a study and showed that TNBC with p63 gene mutations received platinum-based drug-based neoadjuvant therapy and was pathologically complete. The pCR was significantly increased (23 vs 0%, P = 0.048) [43]. However, another phase II clinical trial involving 86 patients with BC showed that platinum-based monotherapy was used in the treatment of first-line or second-line metastatic BC, and patients with p63 gene mutations did not show a higher pCR, RFS, and OS [44].

BRCA-1 gene plays a key role in repairing DNA double-strand damage and maintaining DNA stability by homologous recombination. BRCA-1 mutated tumor cells mainly rely on PARP-mediated DNA repair [45]. Platinum drugs, including cisplatin and carboplatin, kill tumor cells by cross-linking and breaking DNA double strands. This makes tumor cells defective in DNA homologous recombination and highly sensitive to platinum drugs, such as BRCA-1 gene mutant cells. Some TNBCs have inherent genetic instability that leads to DNA repair defects, making them extremely sensitive to chemotherapeutic drugs, such as platinum, that damage the DNA chain and DNA double-strand cross-links and cause replication to stagnate [7]. According to the previous report, about 70% of BRCA-1 gene mutation BC patients are TNBC [46,47]. Patients with TNBC can also carry BRCA-1 gene mutations, showing BRCA-1 gene-deficient tumor gene expression profiles [47,48]. There has been a resurgence of interest in cisplatin in TNBC, in part due to improvements in its side-effect management strategies and additional preclinical data suggesting that platinum may be particularly active in TNBC, as there are histological similarities between BRCA1-mutated BC and TNBC [16]. BRCA-1-related BCs mostly occur in the basal cell-like subtypes of BC and show the gene expression profile of the basal cell-like subtypes. More pieces of evidence indicate that the basal-like subtypes of TNBC mainly pass through BRCA-1-related pathways, leading to increased genome instability [49]. BCs with TNBC and BRCA-1 gene mutations have the same pathological and molecular characteristics, including genome instability, DNA repair defects, and TP53 gene mutations. The basal cell-like subtype of TNBC, whether it has BRCA-1 gene mutation or not, is more sensitive to platinum than other TNBCs [49]. Alba et al. included 93 patients with basal cell-like TNBC in a phase II RCT. The objective response rate of the platinum-containing drug group was higher than that of the non-platinum drug group (76.6% vs 69.6%) [27]. A phase II clinical RCT by Tomasz Byrski et al. conducted a phase II study and showed that the clinical response rate is as high as 80% in those patients with metastatic BC with BRCA-1 gene mutation who received cisplatin monotherapy, and the clinicopathological remission rate is 45% [50]. Similarly, cisplatin monotherapy has a good effect on the neoadjuvant treatment of BRCA-1 gene mutation BC. A prospective trial by Byrski et al. showed that 10 BC patients with BRCA-1 gene mutations received NAC with cisplatin monotherapy, and the pathological complete remission was 90% [51].

There are also some limitations to our study. Firstly, this meta-analysis did not contain the information of individual patients which may result in heterogeneity among studies. Secondly, due to the lack of platinum-associated information, we cannot evaluate the relationship between treatment effect and toxicity. Thirdly, although nine studies included the BRCA mutated TNBC, the data of other mutations were still not enough for assessing the efficacy of platinum in all kinds of TNBC patients. Further studies should be considered for different genetic and pathological types.

## 5. Conclusion

Our systematic review and meta-analysis demonstrated that TNBC patients treated with PBAT could achieve a higher pCR rate and better RFS benefit while without increasing a significantly higher complication rate. However, there needs to be a long time for observation if OS could be improved in the future.
