# Expression of Concern: Platinum-based adjuvant therapy was efficient for triple-negative breast cancer: a meta-analysis from randomized controlled trials

**DOI:** 10.1080/21655979.2026.2667634

**Published:** 2026-05-21

We, the Editors and Publisher of the journal *Bioengineered*, are issuing an Expression of Concern for the following article.

Xie, K., Ren, X., Hong, X., Zhu, S., Wang, D., Ye, X., & Ren, X. (2022). Platinum-based adjuvant therapy was efficient for triple-negative breast cancer: a meta-analysis from randomized controlled trials. *Bioengineered*, 13(6), 14827–14839. https://doi.org/10.1080/21655979.2022.2115616

Following publication, internal integrity audits by the journal and publisher identified concerns about further required methodological details and ethical information.

We have contacted the authors to provide further information, but we have not received a response within the requested timeframe. Therefore, through this Expression of Concern we advise readers to interpret the information presented in the article with due caution.

We have been informed in our decision-making by our editorial policies and COPE guidelines.

The authors have been notified about this Expression of Concern.

